# Platelet subpopulations remain despite strong dual agonist stimulation and can be characterised using a novel six-colour flow cytometry protocol

**DOI:** 10.1038/s41598-017-19126-8

**Published:** 2018-01-23

**Authors:** Anna Linnea Södergren, Sofia Ramström

**Affiliations:** 1 Department of Clinical and Experimental Medicine, Linköping University, Linköping, Sweden; 2 Department of Clinical Chemistry and Department of Clinical and Experimental Medicine, Linköping University, Linköping, Sweden; 3 School of Medical Sciences, Örebro University, Örebro, Sweden

## Abstract

It is recognised that platelets respond differently to activation, where a subpopulation of platelets adopt a procoagulant phenotype while others are aggregatory. However, it has not been thoroughly tested whether these subpopulations will remain in maximally activated samples, or if they are merely a result of different platelet sensitivities to agonist activation. Here platelets were activated with gradually increasing concentrations of thrombin and/or the GPVI agonist cross-linked collagen-related peptide (CRP-XL). Platelet activation was investigated using a novel six-colour flow cytometry protocol evaluating exposure of phosphatidylserine, active conformation of the fibrinogen receptor α_IIb_β_3_, α-granule and lysosomal release (P-selectin and LAMP-1 exposure), mitochondrial membrane integrity and platelet fragmentation. Upon activation by CRP-XL or thrombin+CRP-XL, platelets formed three differently sized subpopulations. Normal-sized platelets showed high exposure of aggregatory active α_IIb_β_3_ and intact mitochondria, while the smaller platelets and platelet fragments showed high exposure of procoagulant phosphatidylserine. The distribution of platelets between the differently sized subpopulations remained stable despite high agonist concentrations. All three were still present after 30 and 60 min of activation, showing that all platelets will not have the same characteristics even after maximal stimulation. This suggests that platelet subpopulations with distinct activation patterns exist within the total platelet population.

## Introduction

Platelets are small cell fragments that play a crucial role in haemostasis. Platelets circulate in a resting state close to the vessel wall, where they may quickly detect breaches in vessel integrity. Upon vessel damage, platelets adhere to collagen and other substances in the subendothelial matrix that become exposed at the site of damage. Adhered platelets become activated and undergo substantial reorganisation with spreading on the surface. Activated platelets release the content of their granules, leading to recruitment of additional platelets to the site of damage, and the formation of a platelet aggregate. Activated platelet will also support the generation of thrombin via the coagulation system. Thrombin will induce further platelet activation, and when present in sufficient amounts generate a fibrin network that reinforce the platelet aggregate. Balance in the haemostatic system is important, as too little activity may result in excessive bleeding, whereas too much activity may lead to vessel occlusion from blood clots.

Platelets were long thought of as being either resting or activated. It was also believed that all activated platelets carried out all platelet functions. However, in the last few decades, the concept of platelet subpopulations has emerged. Recent reviews^1,2^ describe that platelets can take on different roles in haemostasis, either aggregation or support of coagulation.

All platelets may express an active fibrinogen receptor (α_IIb_β_3_) upon activation, which is a requirement for platelet aggregation. On the other hand, flow cytometry studies have demonstrated that only a subset of platelets expose phosphatidylserine (PS) upon strong agonist stimulation^3–5^. Since PS exposure enhance the generation of thrombin by allowing assembly of the prothrombinase complex on the platelet surface, these platelets are considered to have a procoagulant phenotype.

It is generally reported that a high level of platelet activation, surpassing a threshold level in intracellular calcium^2,6^, is needed for the formation of procoagulant platelets. Earlier studies describe a combination of GPVI/FcRγ agonists (such as collagen or convulxin) and thrombin to be the most potent in this regard^3,5,7,8^. However, the postulated potency of thrombin differ, where results vary from a few percent^9–11^ up to 50%^12^ PS-positive platelets. Most studies only show results using one concentration of platelet agonists, especially in cases where dual agonists are used. In fact, it has not been fully explored whether the procoagulant platelets actually represent a distinct platelet subpopulation, or whether they are just the most reactive platelets, being the first to reach a sufficiently high level of activation to undergo this transformation. With this study we therefore aimed to investigate whether the platelet response is in fact dichotomous or if all platelets can reach the threshold level of activation required to become procoagulant, if exposed to high amounts of relevant activating stimuli.

The possibility to analyse all cells in a solution makes flow cytometry a suitable method to investigate platelet subpopulations. Flow cytometry also allows evaluation of several parameters on each cell, which can help to characterize different subpopulations of platelets^13^. Previously mentioned flow cytometry studies do not describe experiments using more than three colours. In this paper, we present a six-colour flow cytometry protocol which allows for the simultaneous evaluation of the platelet activation markers described below, thus reducing the need for indirect comparisons. (I) PAC-1: recognizes the active conformation of the fibrinogen receptor α_IIb_β_3_^14^, a requirement for platelet aggregation. (II) Annexin V: binds to exposed PS^5,8,15^, needed for assembly of the prothrombinase complex on the platelet surface. (III) Retention of DiIC_1_(5): indicates retained mitochondrial membrane potential, loss of which has been suggested to play a role in the formation of procoagulant platelets^6,16^. (IV) Exposure of P-selectin: a sign of α-granule release, a classical marker of platelet activation. (V) LAMP-1 exposure: indicates lysosomal exocytosis. The function of lysosomal exocytosis from platelets is not known, but has been suggested to play a role in e.g. tissue remodelling. Exposure of LAMP-1 may also be used as an alternative marker of platelet activation^17,18^. (VI) Relative platelet size: including platelet fragmentation and formation of platelet microparticles.

With this protocol, we have characterised the appearance and properties of platelet subpopulations in response to increasing concentrations of thrombin and cross-linked collagen-related peptide (CRP-XL). Our findings reveal that PS exposure is restricted to a fraction of platelets, despite strong dual activation, which support the theory that platelet subpopulations do exist.

## Methods

### Materials

The following reagents were from Becton Dickinson (Franklin Lakes, NJ): PAC-1-fluorescein isothiocyanate (FITC; binds active conformation of α_IIb_β_3_^14^), anti-P-selectin-R-phycoerythrin (PE; CD62P, clone: AK4, indicates α-granule release), PE isotype control antibody (Mouse IgG_1_κ), Annexin V-V450 (binds PS^5,8,15^), PE-cyanine7 (PE-Cy7) isotype control antibody (Mouse IgG_1_κ), and anti-lysosomal associated membrane protein (LAMP)−1-PE-Cy7 (CD107a, clone: H4A3^17^. Unlike LAMP-2^19^ and CD63 (LIMP-1)^20^, which are also present in dense granules, LAMP-1 seems to be a specific indicator of lysosomal release. Anti-CD41-PE-Texas Red-X (ECD; clone: P2, binds α_IIb_) was from Beckman Coulter (Brea, CA). Before use, the CD41-ECD antibody was centrifuged at 16,000 g, 4 °C for 20 min to remove debris. The CD41-ECD antibody did not interfere with PAC-1 binding to activated α_IIb_β_3_ (Supplementary Fig. S1).

1,1′,3,3,3′,3′-Hexamethylindodicarbocyanine Iodide (DiIC_1_(5); Molecular Probes, Eugene, OR) was used to measure mitochondrial membrane potential^21,22^, as its fluorescence profile suited our multicolour protocol. Carbonyl cyanide 3-chlorophenylhydrazone (CCCP; Sigma-Aldrich, St. Louis, MO) disrupts the mitochondrial membrane potential^21,22^ and was used as a control for DiIC_1_(5).

Platelets were activated using a cross-linked collagen-related peptide (CRP-XL; Gly-Cys-Hyp-(Gly-Pro-Hyp)10-Gly-Cys-Hyp-Gly-NH2)^23^, purchased from Prof. Richard Farndale, University of Cambridge, UK, human α-thrombin (T6884, Sigma-Aldrich) and/or convulxin (Pentapharm, Basel, Switzerland).

The HEPES buffer had the following composition: 137 mM NaCl, 2.7 mM KCl, 1 mM MgCl_2_, 5.6 mM glucose, 1 g/L BSA, 20 mM 4-(2-Hydroxyethyl)piperazine-1-ethanesulfonic acid (HEPES), pH 7.4^14^. This buffer was used with or without supplementation of 1.5 mM CaCl_2_ (denoted HEPES-Ca^2+^) or 10 mM EDTA (denoted HEPES-EDTA). All chemicals were of reagent grade and from Sigma-Aldrich.

Fibrin polymerization was inhibited by the peptide glycine-proline-arginine-proline (GPRP; JPT peptide Technologies, Berlin, Germany)^24,25^.

### Blood collection

Venous blood was collected from healthy volunteers into 3.2% sodium citrate tubes (Vacuette^®^, Greiner Bio-One, Kremsmünster, Austria). Experiments were started approximately 1 hour after blood collection, as platelet activation responses are more consistent at this time^26^. The procedure was carried out in accordance with current legislation. All participants gave informed consent and the procedure was approved by the regional ethics review board in Linköping, Sweden (Dnr 2012/382–31).

### Flow cytometry

Whole blood was added to a mastermix (1:12 dilution) with the following constituents (all final concentrations): GPRP 2 mM, Ca^2+^ 1.5 mM (to maintain a near physiological Ca^2+^ concentration), agonists (thrombin 0–16.7 U/mL, CRP-XL 0–20 μg/mL, or buffer for controls), and antibodies/probes; anti-CD41-ECD (0.69 μg/mL), PAC-1-FITC (0.56 μg/mL), anti-P-selectin-PE (0.17 μg/mL), anti-LAMP-1-PE-Cy7 (0.5 μg/mL), Annexin V-V450 (2.67 ng/mL), and DiIC_1_(5) (30 nM). Isotype control samples contained HEPES or HEPES-EDTA, GPRP (2 mM) and anti-CD41-ECD (0.69 μg/mL), PAC-1-FITC (0.56 μg/mL), PE isotype control (0.17 μg/mL), PE-Cy7 isotype control (0.5 μg/mL), Annexin V-V450 (2.67 ng/mL), and DiIC_1_(5) (30 nM). CCCP (100 μM) was added to one buffer-stimulated control sample to obtain a fluorescence background for DiIC_1_(5). Following 10-min incubation at room temperature, the labelling was terminated by a 20 times dilution in HEPES-Ca2+ (or HEPES for isotype control samples). Activation at room temperature was used, as described in the first study on whole blood flow cytometry^27^. Comparative studies show that this enhances rather than decreases the platelet activation responses^9,13^.

Analysis on a Gallios flow cytometer (Beckman Coulter) was carried out immediately after dilution and finished within one hour. The flow cytometer was equipped with three lasers (405 nm, 488 nm and 638 nm) and a 10-colour configuration. Acquisition was performed for 90 seconds or until 10,000 platelets had been collected in a temporary platelet gate. To allow for sensitive detection of small platelet-derived particles, we used the ultra-wide angle of detection (submicron particle setting) for forward scatter (FSC) and a fluorescence threshold on FL3 (CD41-ECD) to allow detection of particles otherwise excluded by the FSC threshold^28–30^. Most platelet fragments were only detected when CD41-fluorescence was used to trigger detection, as they appeared below the lowest possible threshold for FSC (Supplementary Fig. S2). Compensation settings were confirmed using conventional and fluorescence-minus-one controls. The flow cytometer performance was checked daily using procedures recommended by the manufacturer. Repeated tests of the flow cytometer acquisition rate showed a CV below 10% for repeated counting of fluorescent beads using the same protocol as for the platelet analysis. To test whether the 1:12-dilution was enough to prevent significant loss of single platelets due to aggregation, we compared acquisition times in resting (n = 30) and thrombin-activated samples (n = 49) in five donors. Acquisition times were 30.7 ± 4.1 vs. 32.6 ± 4.0 s, with a mean difference of 5% between resting and activated samples in the same donor. Although a crude measure, this indicates that substantial loss of platelets as aggregates does not occur. As the whole blood samples also contain leukocytes, although at lower concentrations than platelets, we also investigated whether platelets were lost from analysis due to formation of platelet-leukocyte conjugates. We could confirm that exposure of activation markers did not differ between platelets from whole blood and platelet-rich-plasma from the same donors (p > 0.05, n = 3).

### Data analysis

**Figure 1:**
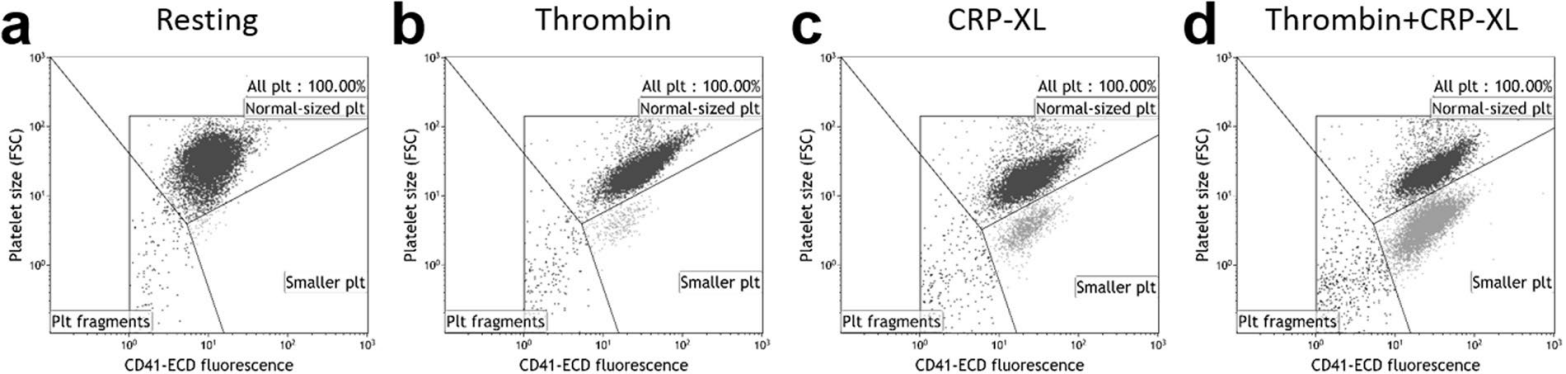
Flow cytometry detect three differently sized platelet subpopulations formed upon platelet activation. Platelets stimulated for 10 minutes with (**a**) buffer (Resting), (**b**) thrombin (5 U/mL), (**c**) cross-linked collagen-related peptide (CRP-XL; 5 μg/mL) or (**d**) thrombin+CRP-XL were investigated using flow cytometry. The figure shows plots of forward scatter (FSC) vs fluorescence intensity of the platelet antibody CD41-ECD and the distribution of platelets between the regions: Normal-sized platelets, Smaller platelets and Platelet fragments, as well as the region for All platelet-derived particles (“All plt”) which was used if subpopulations were not analysed separately. The plots are representative of n = 8–13.

Platelet particles were identified based on size (FSC) and CD41-ECD fluorescence intensity. The boundaries of three regions; named “Normal-sized platelets”, “smaller platelets” and “platelet fragments”, were placed to distinguish the differently sized subpopulations observed (Fig. 1). The regions were checked in each sample and adjusted if needed. To reduce the risk of bias, these gates were not further adjusted during the analysis of platelet activation markers.

Binding of antibodies and probes to platelets was determined as median fluorescence intensity (MFI) or as percentage positive platelets. The boundary between positive and negative platelets was set using a negative control/isotype control sample, where a gate was placed to give 1–2% positive platelets in a fluorescence histogram including all platelets, as previously recommended^31^. The isotype-EDTA sample was used to set the positivity gate for PAC-1, as EDTA disrupts the α_IIb_β_3_ receptor^32^. The isotype sample with HEPES buffer (without calcium) was used to set the positivity gates for P-selectin, LAMP-1 and Annexin V, as the binding of Annexin V to PS is calcium dependent^8,15^. The sample with CCCP was used to set the positivity gate for DiIC_1_(5)^21,22^. Analysis of data was performed using the software Kaluza Analysis, v.1.3 (Beckman Coulter).

### Statistics

Statistical analysis was performed using GraphPad Prism v.5.04 (GraphPad Software, La Jolla, CA). Analysis of variance (ANOVA), or repeated measures ANOVA for paired data sets, followed by Bonferroni’s post-hoc test was performed to compare different treatments. Significance of correlations were calculated using Pearson or Spearman correlation coefficients for parametric and non-parametric data sets, respectively.

### Availability of materials and data

The datasets generated during and/or analysed during the current study are available from the corresponding author on reasonable request.

## Results

### Three differently sized platelet subpopulations are detected upon platelet activation

Using the protocol described above, we observed that resting samples form a single platelet population with roughly equal size, when plotted as FSC/CD41. Strongly activated platelets instead formed three differently sized platelet subpopulations (Fig. 1). A distinct platelet subpopulation (smaller platelets) with lower FSC but relatively similar CD41 expression appeared in activated samples concomitant with an increase in the number of platelet fragments. As both the smaller platelets and these even smaller platelet fragments are likely referred to as “platelet microparticles” in other publications, we chose not to use this term, but to refer to the three subpopulations as “normal-sized platelets”, “smaller platelets” and “platelet fragments”.

Similar to resting platelets, platelets stimulated with thrombin were mainly detected in the region for normal-sized platelets. Platelet activation with CRP-XL increased the fraction of smaller platelets and reduced the fraction of normal-sized platelets. This was further enhanced by the combined use of thrombin+CRP-XL **(**Table 1**)**.

**Table 1:** Fractions of differently sized platelet subpopulations.

Stimulation (10-min)	Normal-sized platelets	Smaller platelets	Platelet fragments
Resting	98.5 ± 0.4%	0.4 ± 0.2%	1.0 ± 0.3%
Thrombin (5U/ml)	97.3 ± 1.0%,	1.7 ± 0.6%,	1.0 ± 0.5%,
CRP-XL (5 μg/ml)	86.8 ± 6.8%	11.4 ± 6.1	1.9 ± 0.9%
Thrombin+CRP-XL	55.7 ± 17.5%	42.1 ± 17.0%	2.3 ± 0.7%

Platelet regions are defined in Fig. 1. Platelets were stimulated with buffer (in resting control samples) or the indicated agonists for 10 min. Mean ± standard deviation, n = 8–13. CRP-XL – Cross-linked collagen-related peptide.

The time required to detect a total of 10,000 normal-sized and smaller platelets showed little variation despite different degrees of fragmentation (mean CV 3.17%, n = 175 samples from 5 donors, with 0–63.4% smaller platelets, Supplementary Fig. S3). This suggests that only one smaller platelet was formed from each normal-sized platelet. Although acquisition time is a rough measure, the sensitivity is sufficient to detect large differences in particle concentration. If each normal-sized platelet would form several smaller platelets, the acquisition time would have been substantially reduced in samples with a high fraction of smaller platelets.

### Differently sized platelet subpopulations have different expression patterns of platelet activation markers

**Figure 2:**
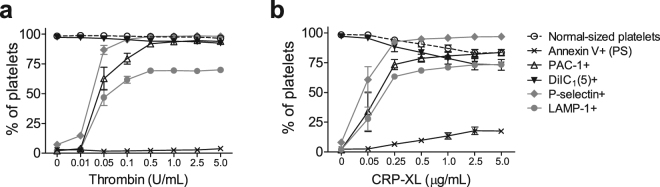
Not all platelet activation markers will become expressed by all platelets even with increasing platelet activation. Platelet samples were stimulated for 10 minutes with increasing concentrations of (**a**) thrombin or (**b**) cross-linked collagen-related peptide (CRP-XL). Platelet activation markers were analysed for the “All plt” region (Fig. 1) and plotted as percentage of platelet-derived particles positioned in the gate for Normal-sized platelets, and as percentage of all platelet-derived particles positive for Annexin V (Annexin V+), indicating phosphatidylserine (PS) exposure, PAC-1+, indicating active conformation of α_IIb_β_3_, DiIC_1_(5)+, where a positive signal indicates retention of mitochondrial membrane potential, P-selectin+, indicating α-granule exocytosis, and LAMP-1+, indicating lysosomal exocytosis. The graph shows mean values with standard error of the mean (SEM), n = 5.

Next, we investigated if and how the expression of platelet activation markers differed between normal-sized platelets, smaller platelets and platelet fragments as compared to “all platelet-derived particles” (“All plt” in Fig. 1). Figure 2 shows how the different platelet activation markers changed in all platelets upon stimulation with increasing concentrations of thrombin (Fig. 2a) and CRP-XL (Fig. 2b). Both agonists gave a similar pattern for PAC-1 binding (active α_IIb_β_3_) and for the granule release markers P-selectin (α-granules) and LAMP-1 (lysosomes). P-selectin was the most sensitive activation marker at low agonist concentrations, followed by PAC-1 and LAMP-1, which all levelled out at the same agonist concentrations. The fraction of LAMP-1 positive platelets levelled out at approximately 70%, and never reached 100%. Stimulation with CRP-XL resulted in a concentration-dependent decrease in the fraction of normal-sized platelets. This decrease seemed to correspond to an increase in Annexin V-binding platelets and a decrease in DiIC_1_(5) fluorescence. The final level of PAC-1 binding platelets was lower in platelets stimulated with CRP-XL compared to thrombin (Fig. 2b). In addition to CRP-XL, platelets were also activated with the snake venom convulxin, which acts via the GPVI receptor (Supplementary Fig. S4). The results with CRP-XL and convulxin were similar, therefore we decided to continue with CRP-XL, as this agonist more closely resembles the physiologic agonist collagen.

**Figure 3:**
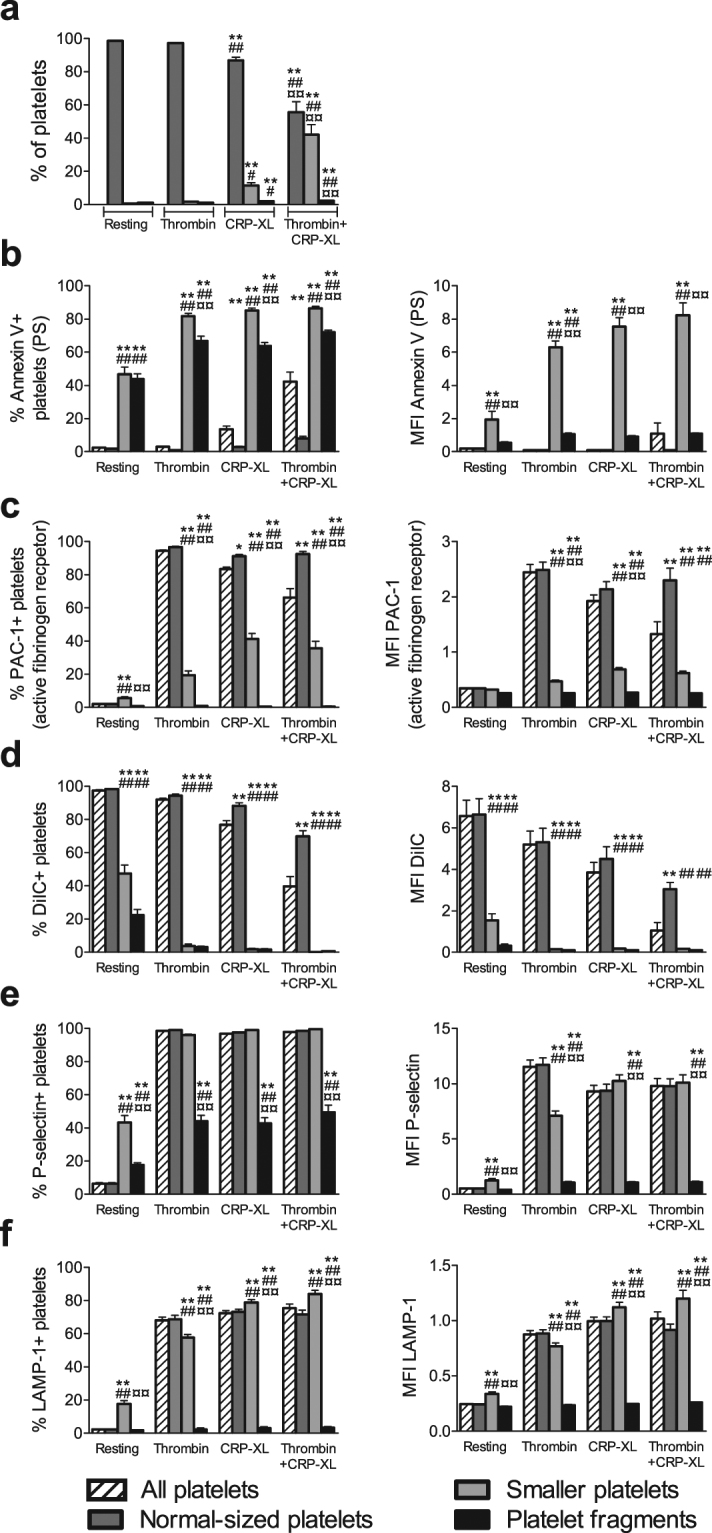
Platelet subpopulations show distinct differences in exposure of platelet activation markers after 10 minutes of platelet activation. Platelet samples were stimulated for 10 minutes with buffer (resting), thrombin (5 U/mL), cross-linked collagen-related peptide (CRP-XL; 5 μg/mL) or thrombin+CRP-XL. (**a**) Distribution of platelet particles between the regions for normal-sized platelets, smaller platelets and platelet fragments (defined in Fig. 1). ^*/#/¤^p < 0.05, ^**/##/¤¤^p < 0.01 as compared to resting platelets/thrombin/CRP-XL. (**b**–**f**) Exposure of platelet activation markers in all platelets and the differently sized platelet subpopulations, determined as percentage of positive platelets (left column) and median fluorescence intensity (MFI; right column) of (**b**) Annexin V, indicating phosphatidylserine (PS) exposure, (**c**) PAC-1, indicating active conformation of α_IIb_β_3_, (**d**) DiIC_1_(5), where fluorescence indicates retention of mitochondrial membrane potential, (**e**) P-selectin, indicating α-granule exocytosis and (**f**) LAMP-1, indicating lysosomal exocytosis. ^*/#/¤^p < 0.05, ^**/##/¤¤^p < 0.01 as compared to All platelets/Normal-sized platelets/Smaller platelets. Bars indicate mean value and standard error of the mean (SEM), n = 8–13. Note that differences for smaller platelets and fragments in resting and thrombin-activated samples should be interpreted with caution due to the low numbers in these samples.

To further characterize the different platelet subpopulations, we studied the exposure of platelet activation markers on the three differently sized platelet subpopulations that formed upon exposure to thrombin (5 U/mL), CRP-XL (5 μg/mL) or thrombin+CRP-XL (Fig. 3). Annexin V binding was almost exclusively found on smaller platelets and platelet fragments (Fig. 3b), whereas PAC-1 mainly bound to normal-sized platelets (Fig. 3c).

DiIC_1_(5) fluorescence was detected in normal-sized platelets, but was essentially absent in smaller platelets and platelet fragments (Fig. 3d). The exposure of P-selectin and LAMP-1 (Fig. 3e–f) did not vary much between normal-sized platelets and smaller platelets. There was a trend towards a slightly higher exposure on smaller platelets in samples stimulated with CRP-XL or thrombin+CRP-XL, although it was only significant for LAMP-1. For all agonists, the exposure of P-selectin and LAMP-1 was significantly lower in platelet fragments than in normal-sized and smaller platelets. As the fluorescence for P-selectin and LAMP-1 in platelet fragments was low, we tested whether a specific signal could be detected by comparing the specific and isotype antibody fluorescence in these. This showed that a specific increase in P-selectin-PE fluorescence could be detected, whereas this was not possible for LAMP-1. Thus, reliable detection of LAMP-1 exposure in platelet fragments was not possible (Supplementary Fig. S5).

When activation was increased, the fluorescence of DiIC_1_(5) in normal-sized platelets decreased, especially when studying thrombin+CRP-XL (Fig. 3d). Therefore, we performed a substudy comparing the expression of other activation markers between normal-sized platelets positive and negative for DiIC_1_(5) fluorescence. For DiIC_1_(5)-negative platelets the fraction of Annexin V-positive platelets was higher, but PAC-1, P-selectin and LAMP-1 was lower (p < 0.01, Supplementary Fig. S6). These platelets also showed lower FSC and CD41 fluorescence than the DiIC_1_(5)-positive ones. The changes were however small as compared to the smaller platelets.

### Formation of differently sized platelet subpopulations level out at higher agonist concentrations

**Figure 4:**
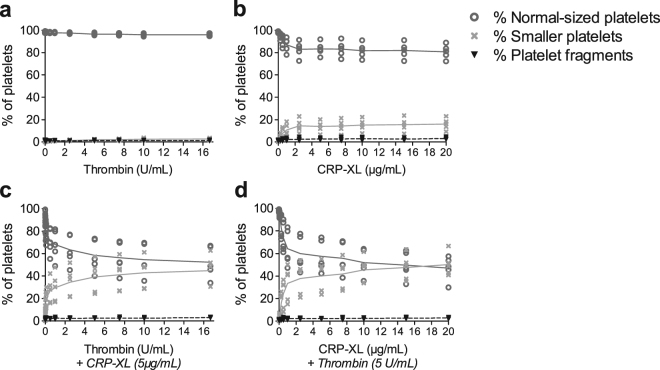
The fraction of platelets distributed in the regions of normal-sized platelets, smaller platelets and platelet fragments do not change further with increasing concentrations of platelet agonists. Platelets were stimulated for 10 minutes with (**a**) thrombin (0–16.7 U/mL), (**b**) cross-linked collagen-related peptide (CRP-XL; 0–20 μg/mL), (**c**) thrombin (0–16.7 U/mL)+CRP-XL (5 μg/mL) or (**d**) CRP-XL (0–20 μg/mL)+thrombin (5 U/mL). The line indicates the mean and the dots indicate samples from different donors (n = 5). Platelet regions are defined in Fig. 1.

As none of the conditions examined above could turn all platelets into smaller platelets or positive for Annexin V binding, we investigated whether even higher agonist concentrations could do this. However, the distribution of platelets between the differently sized platelet subpopulations reached a plateau (Fig. 4a,b). The fraction of normal-sized platelets was unaffected by agonist stimulation above 5 U/mL thrombin or 2.5 μg/mL CRP-XL (Supplementary Fig. S7). When a maximally activating concentration of CRP-XL or thrombin was combined with increasing concentrations of thrombin or CRP-XL, respectively (Fig. 4c,d), the degree of platelet fragmentation increased compared to single agonists, but the percentage of normal-sized platelets levelled out at a combination of 5 U/mL thrombin+5 μg/mL CRP-XL (Supplementary Fig. S7). Similar results were obtained when platelets were activated with convulxin with or without addition of thrombin (Supplementary Fig. S4). An individual variation in the donors’ ability to form subpopulations was found, where the fraction of smaller platelets ranged from 19–66% at 5 U/mL thrombin+5 μg/mL CRP-XL. All donors followed the same trend throughout the concentration span (Fig. 4c,d).

**Figure 5:**
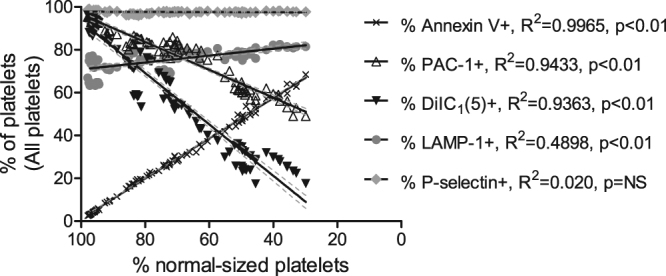
Exposure of platelet activation markers is strongly correlated to platelet fragmentation. Correlation of platelet fragmentation (seen as % normal-sized platelets remaining, note the reversed scale on the axis) versus the percentage of all platelets (“All plt” region in Fig. 1) positive for different activation markers, i.e. phosphatidylserine (Annexin V), active conformation of α_IIb_β_3_ (PAC-1), mitochondrial membrane potential (DiIC_1_(5)), lysosomal exocytosis (LAMP-1) and α-granule secretion (P-selectin). Data points plotted are from samples stimulated with high concentrations of thrombin (5–16.7 U/mL) and/or CRP-XL (5–20 μg/mL) for 10 minutes. n = 90 from 10 independent experiments. The lines show the linear regression with 95% confidence interval for the different data sets.

Some platelet activation markers showed a very strong correlation to the decrease in normal-sized platelets, which occurred when the fraction of smaller platelets and platelet fragments increased (Fig. 5). Binding of PAC-1 and DiIC_1_(5) fluorescence decreased, while Annexin V-binding increased. LAMP-1 exposure showed a weak inverse correlation, whereas P-selectin exposure showed no correlation to the fraction of normal-sized platelets.

We also investigated whether the normal-sized platelets retained their expression patterns at even higher agonist concentrations. For single agonists, no significant differences in expression were observed using concentrations above 2.5 U/mL thrombin or 2.5 μg/mL CRP-XL (Supplementary Fig. S7). For dual agonists, no significant differences in MFI values were observed when applying concentrations higher than 5 U/mL thrombin+5 μg/mL of CRP-XL. The same was true for the fraction of positive platelets, except for Annexin V and DiIC_1_(5), which showed slight changes up to 7.5 U/mL thrombin+5 μg/mL CRP-XL or 10 μg/mL CRP-XL+5 U/mL thrombin.

### Expression patterns on differently sized platelet subpopulations after prolonged incubation times

**Figure 6:**
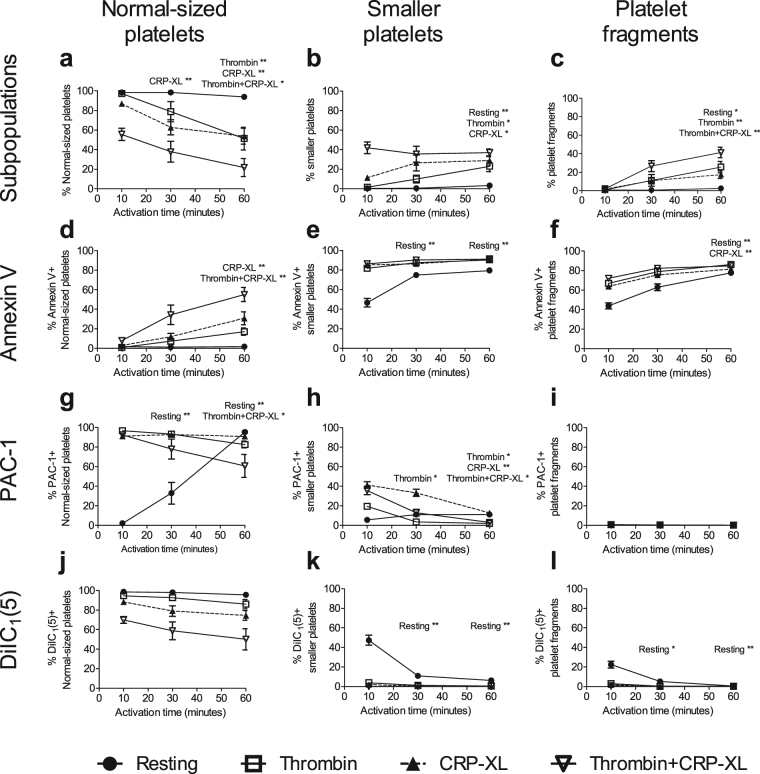
Additional but slightly different platelet subpopulations form with prolonged activation times. Platelet samples were stimulated with buffer (Resting; black circles), thrombin (white squares; 5 U/mL), cross-linked collagen-related peptide (CRP-XL; 5 μg/mL, black triangles, base down, broken line) or thrombin+CRP-XL (white triangles, base up). The graphs show (**a**–**c**) formation of the differently sized platelet particles (defined in Fig. 1) and (**d**–**l**) exposure of platelet activation markers as percentage positive platelets in the differently sized platelet subpopulations after 10, 30 and 60 minutes’ activation. (**d**–**f**) Annexin V, indicating phosphatidylserine exposure, (**g**–**i**) PAC-1, indicating active conformation of α_IIb_β_3_, and (**j**–**l**) DiIC_1_(5), where colour indicates retention of mitochondrial membrane potential. Graphs show mean value and standard error of the mean (SEM), n = 5–13. *p < 0.05, **p < 0.01 as compared to the corresponding result after 10 minutes’ activation. In resting samples, the number of smaller platelets and platelet fragments are low, hence these data should be interpreted with caution.

To investigate whether all platelets would be able to take on the same phenotype over time, we increased the time of platelet activation to 30 and 60 min. Notably, after this time, platelets showed a high degree of pre-activation with exposure of P-selectin (54 ± 34% and 97 ± 1% after 30 and 60 min respectively, mean ± standard deviation) and PAC-1 binding also in the “resting” control samples (Fig. 6g). The fraction of normal-sized platelets decreased by prolonged incubation with both thrombin and CRP-XL, accompanied by an increase in smaller platelets and platelet fragments (Fig. 6a–c and Supplementary Fig. S8), but still there were normal-sized platelets present. Notably, at 60 minutes, the fraction of normal-sized platelets was similar for CRP-XL and thrombin. However, thrombin-activated normal-sized platelets still showed higher DiIC_1_(5) fluorescence and lower Annexin V binding (Fig. 6j,d, respectively).

The percentage of Annexin V-positive normal-sized platelets was significantly increased at 60 min when exposed to CRP-XL and thrombin+CRP-XL (Fig. 6d), but the MFI (Supplementary Fig. S9) remained low at all time points. The same trend, although not significant, could be seen using thrombin as agonist. Fewer changes were seen on smaller platelets and platelet fragments. For smaller platelets, a significant decrease in Annexin V MFI was seen in platelets stimulated with thrombin+CRP-XL for 60 min. Moreover, 60 min stimulation with CRP-XL increased the fraction of Annexin V-positive fragments (Fig. 6f). PAC-1 binding to the smaller platelets was further reduced with time (Fig. 6h). For DiIC_1_(5), prolonged activation induced no significant changes in staining in any of the activated platelet samples (Fig. 6j–l).

## Discussion

Multi-colour flow cytometry protocols are uncommon in platelet research. Our aim was to develop and present such a protocol, including a broad range of platelet activation markers. Using this protocol, we investigated whether platelets form distinct subpopulations upon agonist-induced activation, and the activation markers that characterized these. We chose to focus on platelet responses after 10 minutes of activation, to reflect the initial platelet response upon contact with the damaged vessel. This time point has been shown to be more than enough to reach a plateau in platelet procoagulant responses^33,34^. However, prolonged activation times were also included, to enable comparison with other studies, but also to push the formation of platelet subpopulations maximally. Indeed, platelets located in a thrombus will remain there for some time. Thrombus remodelling or embolization during this period could then make platelet responses after prolonged activation relevant.

In the flow cytometer, resting platelets appear as a single platelet population, here referred to as normal-sized platelets. Platelet activation via GPVI resulted in the formation of two additional platelet subpopulations with smaller size, here called smaller platelets and platelet fragments. In previous publications, the term platelet microparticles has often been used for everything smaller than normal-sized platelets. Thus, to avoid confusion and erroneous comparisons with other studies, we chose to avoid that term and use the terms smaller platelets and platelet fragments in the current study. Differences in instrument sensitivities and settings might explain why we found two additional platelet subpopulations where most studies show only one. Bode and Hickerson demonstrated that the number of smaller particles detected by flow cytometry is not high enough for them to be microparticles and thus postulated that these are smaller platelets^28^. Further, both Bode *et al*.^28^ and Tochetti *et al*.^29^ report that applying a threshold on fluorescence instead of FSC reveals a third subpopulation of much smaller platelet-derived particles, which support our observations. Indeed, with close examination of one of the first platelet subpopulation reports^3^, it seems probable that the subpopulation reported as microparticles is actually the one we detected as smaller platelets. A more recent report also demonstrate a decreased side scatter for the PS-exposing platelets^10^.

The similar exposure patterns of smaller platelets and platelet fragments (discussed further below) indicated that these subpopulations had the same origin. Further, the time required to collect 10,000 platelets in a combined region for normal-sized and smaller platelets was not affected by the degree of platelet fragmentation. This leads us to hypothesize that each smaller platelet is generated from one highly activated normal-sized platelet through the release of platelet fragments.

Our fluorescence trigger approach allowed us to detect platelet fragments with forward scatter below the threshold commonly applied in other studies. Antigen density decreases rapidly with particle size^35^ and has been highlighted as a potential problem for microparticle detection^36^. Thus, we chose the most abundant platelet receptor α_IIb_β_3_ for detection of platelet-derived particles. The fractions of platelet fragments were still rather low, indicating that even this approach miss a substantial part of the platelet fragments.

It is important to emphasize that in the current study, platelet size and exposure of activation markers are determined after platelet activation (in resting samples, all platelets are normal-sized). Therefore, the presented results cannot be compared to previous studies that have investigated activation parameters based on the size of platelets before activation^37–39^.

Exposure of PS, which is detected through binding of Annexin V, is considered to be a marker for a procoagulant platelet phenotype^1,2^. The aggregatory platelet phenotype can instead be detected by binding of the antibody PAC-1^1^. PAC-1 recognizes the active conformation of α_IIb_β_3_ needed for high affinity binding of fibrinogen. After platelet activation, the normal-sized platelets bound PAC-1 but not Annexin V to any substantial degree. Thus, these platelets can be considered proaggregatory. In addition, the normal-sized platelets showed DiIC_1_(5) fluorescence, indicating retained mitochondrial membrane potential. Our study shows that the procoagulant platelets are mainly found among the smaller platelets and platelet fragments, who both bound Annexin V. The smaller platelets and platelet fragments did not bind PAC-1 and had low DiIC_1_(5) fluorescence.

The reduction in PAC-1 binding found on PS-positive platelets has been reported previously and has been described as a down-regulation of the fibrinogen receptor^40^. However, it was recently suggested that this interpretation might need to be revisited^41^, as PS-positive, PAC-1-negative platelets may still bind another conformation-specific antibody, LIBS-6, that also recognize α_IIb_β_3_ when bound to fibrinogen^42^. As PAC-1 has higher affinity for active α_IIb_β_3_ than fibrinogen^14,42^ and was present during the 10 min activation, PAC-1 should have been able to bind a number of α_IIb_β_3_ receptors whenever these became active. Further, the availability of fibrinogen is equal to all platelets in our experiments. Thus, the observed decrease in PAC-1 binding in the Annexin V-positive platelets probably reflects some type of conformational change in the receptor. From this we cannot conclude whether this affects the fibrinogen content on the platelet surface.

The results presented above show that normal-sized platelets have a different exposure pattern than smaller platelets and platelet fragments. In support of this, Fig. 5 shows that the appearance of Annexin V-positive platelets was extremely well correlated to a decrease in the fraction of normal-sized platelets. Thus, in situations where platelet fragmentation is substantial, the activation responses obtained for “all platelets” may not match the results for either normal-sized platelets, smaller platelets or platelet fragments. As an example, platelet activation with thrombin+CRP-XL resulted in Annexin V-binding in 42 ± 6% (mean ± SEM) of “all platelets”. If analysed in the differently sized subpopulations, the results were drastically different, with Annexin V-binding in 9 ± 1% of normal-sized platelets, 86 ± 1% of smaller platelets and 72 ± 1% of platelet fragments. These discrepancies add an extra level of complexity to the analysis of platelet flow cytometry data that could impact on the results and therefore may need to be taken into account.

All platelets had a high expression of the general activation marker P-selectin and the less studied marker LAMP-1^18,43^, indicating that they exhibited a similar degree of granule release. Thus, the differences in exposure were not due to one subpopulation being generally less active. P-selectin expression was the most sensitive sign of low level platelet activation, but LAMP-1 expression and PAC-1 binding showed similar curves. However, LAMP-1 expression was never measurable in 100% of the platelets. Lysosomal exocytosis did not seem to be a specific characteristic, or even required for the formation of the PS-positive smaller platelets, as the LAMP-1 exposure was relatively similar in normal-sized and smaller platelets. The inability to reach 100% LAMP-1 positive platelets could potentially be explained by the low number of lysosomes in platelets (0–3 lysosomes per platelet^44^). Even though the platelet fragments and smaller platelets showed similar patterns for other activation markers, P-selectin and LAMP-1 expression was lower on the fragments. This might be due to their small size, as this reduces the number of antigens available for antibody-based detection^35^. For LAMP-1, our data suggest that the number of antigens on platelet fragments may be reduced below the limit needed for reliable and quantifiable detection.

Activated normal-sized platelets showed a somewhat decreased DiIC_1_(5) fluorescence without a decrease in PAC-1 and increase in Annexin V-binding. This suggests that platelets may lose mitochondrial membrane potential without or before these changes occur. One major advantage of our six-colour protocol is that it allows direct comparison of several activation markers on platelets without running separate experiments. We used this to evaluate differences in DiIC_1_(5)-positive and -negative normal-sized platelets. Considering PAC-1, Annexin V and FSC, the DiIC_1_(5)-negative normal-sized platelets showed signs of being in a transition state, but the changes were still marginal as compared to the drastic changes in smaller platelets (Supplemental Fig. S4). This supports previous reports showing that disruption of mitochondrial potential could be reversible^16^ and occurs before PS exposure^6,16^. This is also supported by the slight decrease in DiIC_1_(5) fluorescence we observed in thrombin-stimulated platelets, without changes in PAC-1 or Annexin V-binding.

Thrombin and GPVI agonists such as CRP-XL and convulxin are the most potent platelet agonists and commonly used to generate procoagulant platelets. Therefore, these agonists were also used in this study. Dose responses of single agonists showed that all agonists were able to activate platelets and give a high exposure of P-selectin, LAMP-1 and binding of PAC-1. CRP-XL (above 0.5 μg/ml) or convulxin (above 10ng/ml) was able to transform a small fraction of platelets from normal-sized, aggregatory platelets into smaller platelets binding Annexin V. The formation of platelet subpopulations did not increase at agonist concentrations above 2.5 μg/ml of CRP-XL or 500 ng/ml of convulxin. In contrast, thrombin showed low potency to induce Annexin V-positive platelets after 10 min stimulation. This might be surprising to some, however, this is the case in most previous studies^5,11,33,45^. Previous publications reporting pronounced PS exposure with thrombin alone frequently use longer incubation times^12,46–48^. In line with these results, we also observed increased Annexin V-binding upon prolonged incubation with thrombin.

The potentiating effect of thrombin on top of a GPVI agonist is previously known^3,5,7^. What has been lacking are experiments using two platelet agonists in gradually increasing concentrations, to confirm or reject the subpopulation hypothesis. When conducting these experiments, we found that the formation of Annexin V-positive smaller platelets reached a plateau, despite very high agonist concentrations. This indicates that only a subset of platelets will take on the procoagulant platelet phenotype upon initial activation. It is also interesting that the donors showed such big differences in the fraction of platelets taking on this phenotype. This supports a previous report, where healthy donors showed a variable but consistent ability to form the PS-positive platelets called coated platelets^49^.

It has been discussed whether the procoagulant platelet response occurs through an apoptotic or necrotic process^50^. With 10 min incubation, a possible mediator for the observed platelet responses is the cyclophilin D-dependent necrotic pathway, as recently reviewed^41^. However, with longer incubation times, we observed other changes, such as increased Annexin V-binding in normal-sized platelets but with retained PAC-1 binding, and a marked increase in smaller platelets and platelet fragments, especially in samples activated by thrombin alone. The MFI, however, indicates that this Annexin V-binding was on a low level. The MFI for Annexin V in smaller platelets also decreased with time. It has been described that PS exposure is lower in apoptotic platelets than in agonist-stimulated platelets^51^. Further, the calcium threshold for necrosis is higher than for apoptosis^41^. This might suggest that during the initial 10 min, Annexin V-positive platelets were mainly formed through a necrotic process, causing high PS exposure. With prolonged stimulation times, smaller platelets would also be generated through an apoptotic process, yielding less PS exposure. Interestingly, several studies using thrombin alone report on events associated with apoptosis^47,48^. Based on this, it could be postulated that thrombin induce PS-exposure mainly through the apoptotic route, whereas CRP-XL may engage both the necrotic and the apoptotic route. Future studies might reveal how these results relate to a recent study reporting platelet subpopulations with or without transglutaminase activity^52^.

In conclusion, we have designed a novel six-colour protocol for platelet flow cytometry. Using this protocol, we report the formation of three distinct platelet subpopulations upon activation. Smaller platelets and platelet fragments exhibited a procoagulant phenotype, with bound Annexin V but not PAC-1, and little DiIC_1_(5) staining. These appeared after activation with GPVI agonists CRP-XL or convulxin or one of these in combination with thrombin, whereas thrombin alone was ineffective. The plateau that formed at higher agonist concentrations indicates that only a fraction of the platelets will initially take on a procoagulant phenotype, while others never reach this state, supporting the theory of platelet subpopulations. At longer incubation times, thrombin however became more effective, suggesting that subpopulation formation may occur through several mechanisms.

## Electronic supplementary material

Supplementary figures
