# Supplementary material for: Platelet subpopulations remain despite strong dual agonist stimulation and can be characterised using a novel six-colour flow cytometry protocol
Source: Sci Rep. 2018 Jan 23;8:1441. doi: 10.1038/s41598-017-19126-8 (PMC5780418; doi:10.1038/s41598-017-19126-8)

Supplementary information

Platelet subpopulations remain despite strong dual agonist stimulation and can be characterised using a novel six-colour flow cytometry protocol

Anna Linnea Södergren[1], Sofia Ramström[2,3]*

[1] Department of Clinical and Experimental Medicine, Linköping University, Linköping, Sweden.
Anna Södergren

[2] Department of Clinical Chemistry and Department of Clinical and Experimental Medicine, Linköping University, Linköping, Sweden.
Sofia Ramström

[3] School of Medical Sciences, Örebro University, Örebro, Sweden
Sofia Ramström

Supplementary Figure S1: PAC-1 binding is possible in the presence of CD41-ECD. Histograms showing PAC-1-FITC (FL-1) fluorescence in samples with

a) Resting platelets in the presence of CD41-ECD (0.69 μg/mL)
b) Platelets activated with cross-linked collagen-related peptide (CRP-XL, 2.5 μg/mL)
c) Platelets activated with CRP-XL (2.5 μg/mL) in the presence of CD41-ECD (0.69 μg/mL)

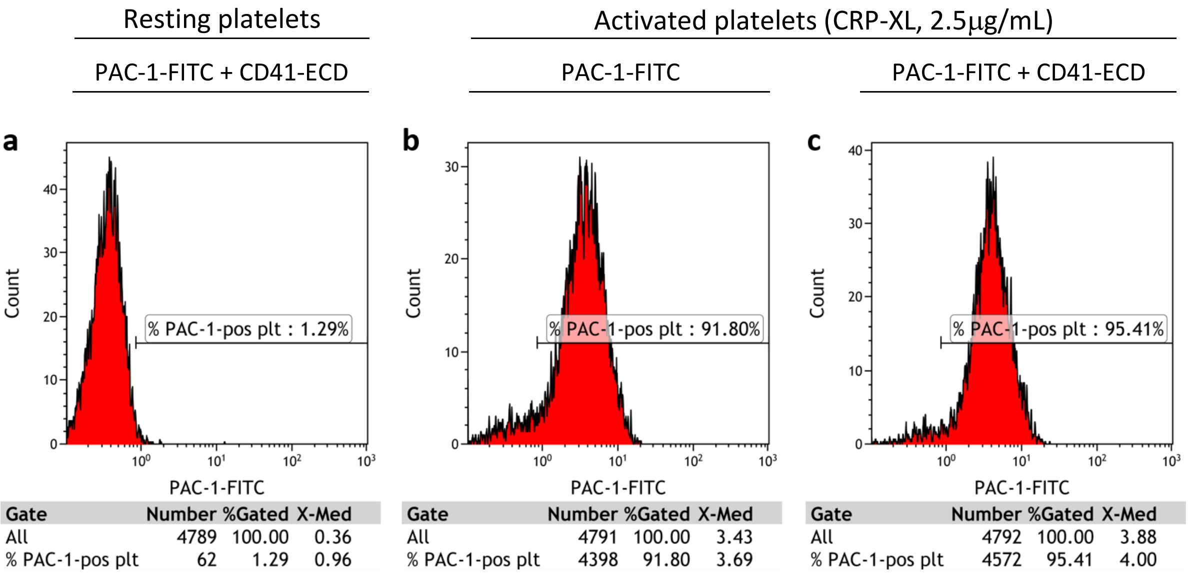

Supplementary information

Södergren AL and Ramström S: *"Platelet subpopulations remain despite strong dual agonist stimulation and can be characterised using a novel six-colour flow cytometry protocol"*

Supplemental Figure S2: Use of a threshold on CD41-ECD reduces background and allows detection of a third population of platelet-derived particles. Comparison between platelet particles detected using a threshold on either forward scatter (the two columns to the left) or fluorescence of the CD41-ECD antibody (FL3) (the two columns to the right). The first and third column shows forward scatter (FSC) vs. side scatter (SSC), with regions for platelets and everything with smaller size as "microparticles". The second and fourth column shows FSC vs. CD41-ECD fluorescence of all particles falling into the "All platelet particles" region in the FSC vs SSC plot, i.e. all particles with a FSC lower than the red and white blood cells. The FSC vs CD41-ECD plot contains regions dividing platelets into normal sized platelets, smaller platelets and platelet fragments. The first row shows particles detected in samples without blood added, the middle row shows results for resting platelets and the bottom row shows results with activated platelets (10 minutes' activation with cross-linked collagen-related peptide (CRP-XL) (2.5 μg/mL) and PAR1-activating peptide (SFLLRN, 30 μM; JPT peptide Technologies, Berlin, Germany)). The acquisition finished after collection of 10,000 particles in the temporary platelet gate (after 20-35 seconds) or after 2 minutes if this criterion was not met (for samples without blood added).

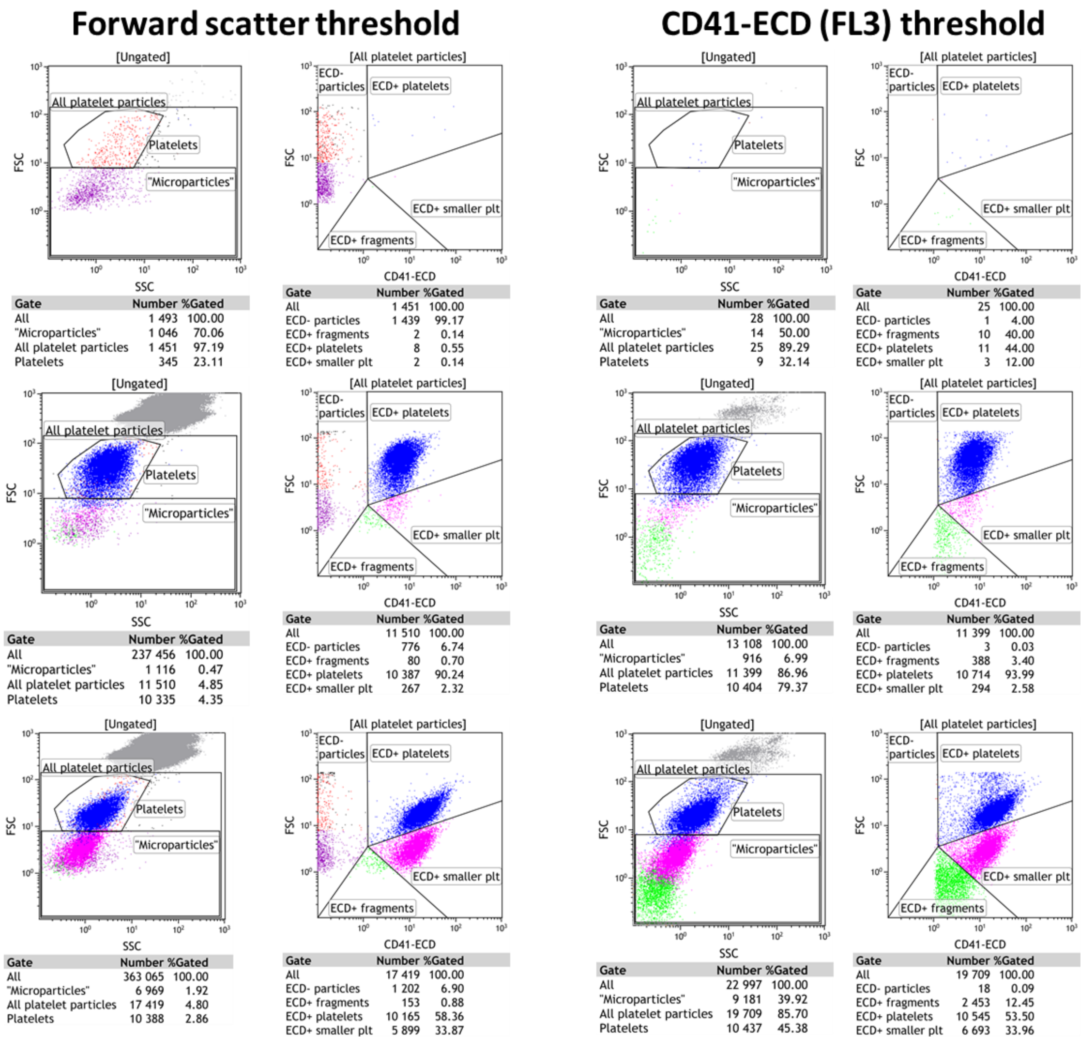

Supplementary information

Södergren AL and Ramström S: *"Platelet subpopulations remain despite strong dual agonist stimulation and can be characterised using a novel six-colour flow cytometry protocol"*

Supplemental Figure S3: The smaller platelets are not platelet microparticles. Correlation between the percentage of "smaller platelets" and the time to detect 10,000 particles in a combined region for normal-sized platelets and smaller platelets (defined in Figure 1). The samples include both resting samples and samples activated for 10 minutes with high concentrations of thrombin and cross-linked collagen-related peptide (CRP-XL; 0-16.7 U/mL thrombin + 5 μg/mL CRP-XL or 0-20 μg/mL CRP-XL + 5 U/mL thrombin; same data as in figure 4 C-D, including 5 donors and 175 samples in total). Statistical analysis reveals a lack of correlation and a very low coefficient of variation (CV) for the time required to detect 10,000 particles in the combined region. This opposes the assumption that "smaller platelets" would be several microparticles released by each activated platelet. If the "smaller platelets" would be microparticles, the time to detect a set number of particles in these two regions should decrease when the fraction of smaller platelets increased.

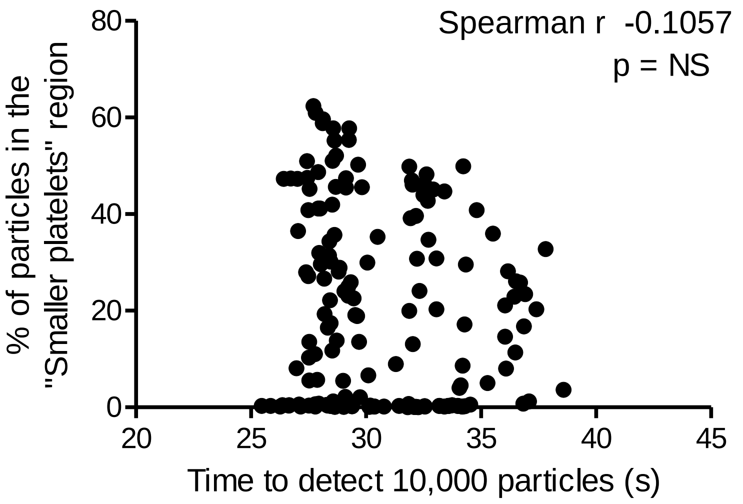

Donor	No. of analysed samples	% smaller platelets (median (range))	Time to detect 10,000 normal-sized and smaller platelets (seconds) (median (range))	CV (%)
1	35	20.0 (0.03-49.9)	32.2 (30.8-34.2)	2.27%
2	35	19.2 (0.2-51.0)	27.9 (25.5-29.5)	3.77%
3	35	8.1 (0.2-40.9)	28.3 (26.7-29.5)	2.61%
4	35	5.0 (0.05-40.8)	34.8 (32.3-38.6)	4.47%
5	35	25.0 (0.09-62.4)	29.3 (27.7-30.5)	2.73%

Supplemental Figure S4: Platelet responses level out despite strong activation with GPVI agonist convulxin. Platelet samples were stimulated for 10 minutes with increasing concentrations of convulxin with or without the addition of thrombin (5U/ml). (a) Platelet activation markers were analysed for the "All plt" region (Fig. 1) and plotted as percentage of platelet-derived particles positioned in the gate for Normal-sized platelets, and as percentage of all platelet-derived particles positive for Annexin V, PAC-1, DiIC$_1$(5), P-selectin and LAMP-1. The graph shows mean values with standard error of the mean (SEM). (b-c) The line indicates the mean and the dots indicate samples from different donors. Platelet regions are defined in Fig. 1. n=5 for all graphs.

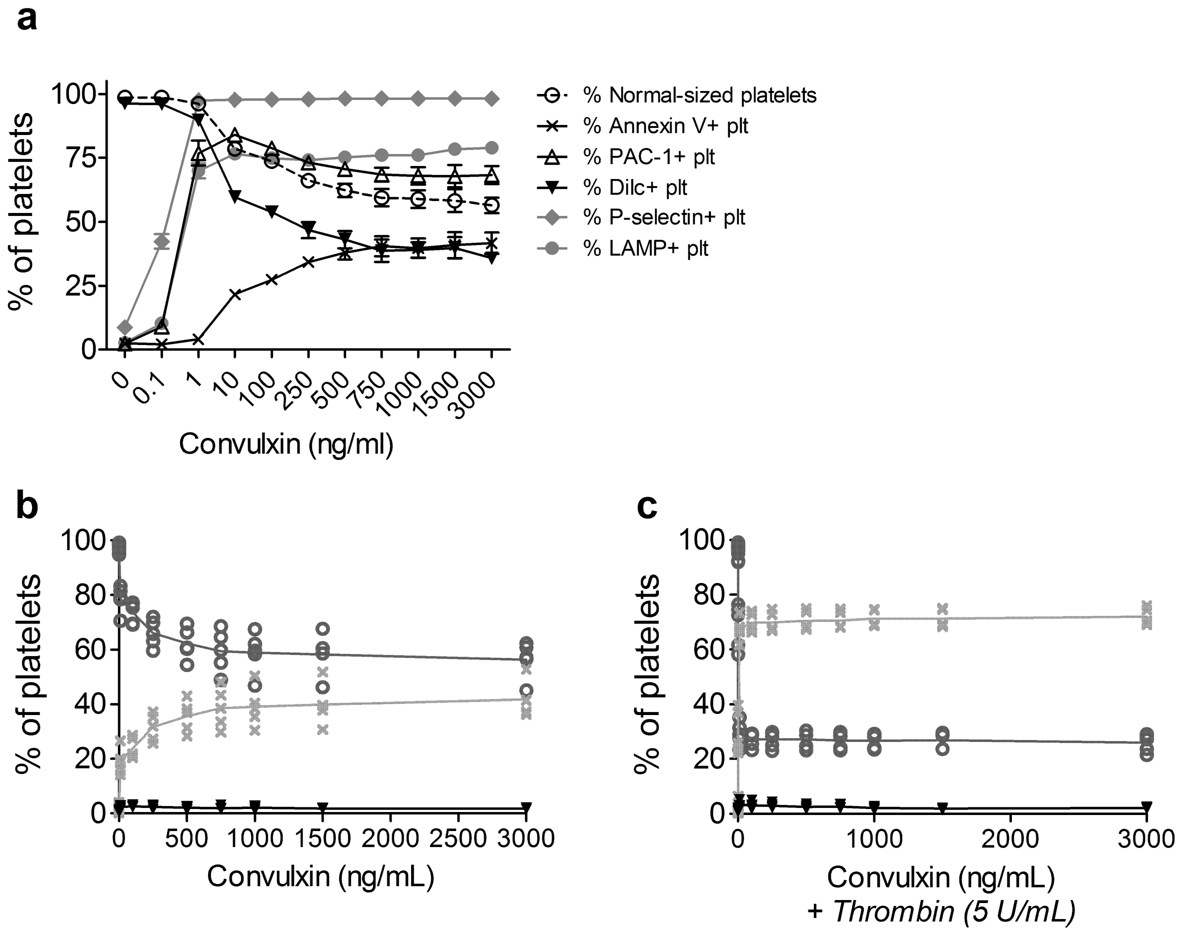

Supplemental Figure S5: P-selectin but not LAMP-1 exposure can reliably be detected on platelet fragments. Median fluorescence intensity (MFI) for (a) anti-P-selectin-PE and PE-isotype or (b) anti-LAMP-1-PE-Cy7 and PE-Cy7-isotype was compared for platelet fragments. MFI for the anti-P-selectin-PE antibody was higher in platelet fragments in activated samples compared to resting samples and PE-isotype. For LAMP-1 detection MFI remained low in all samples indicating that specific detection was not possible. Measurements were performed as described in the manuscript. Briefly venous blood was collected into sodium citrate tubes (Vacutainer®, Becton Dickinson). Platelet were labelled with anti-CD41-PE-Texas Red-X (ECD) and anti-P-selectin-PE and anti-LAMP-1-PE-Cy7 or PE-isotype control antibody and PE-Cy7-isotype control antibody. Platelets were stimulated with thrombin (5U/ml) + CRP-XL (5µg/ml) or buffer for resting control for 10 min at room temperature. Antibody binding was determined by flow cytometry. Lines indicate mean values, dots represent individual samples, n=4.

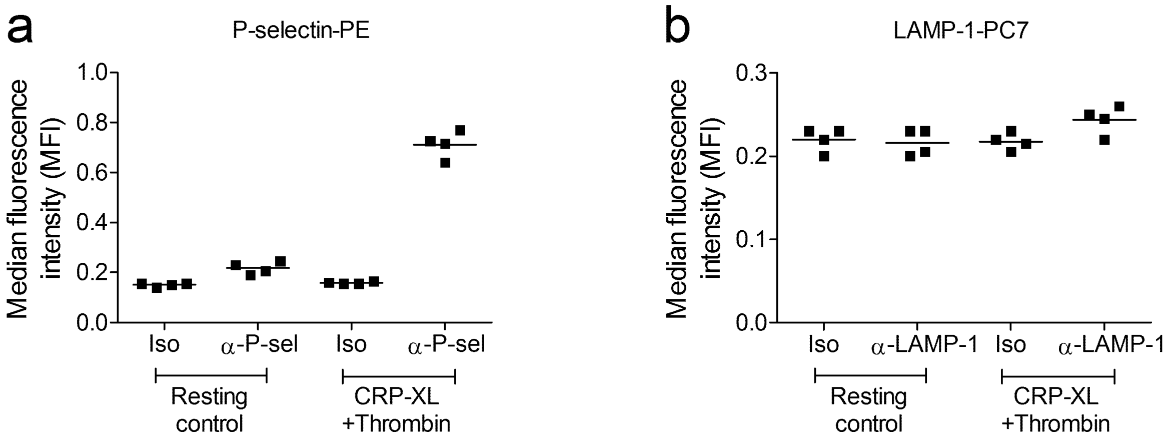

Supplemental Figure S6: Platelet activation markers differ between normal-sized platelets positive and negative for DiIC$_1$(5). Platelet samples were activated with thrombin and cross-linked collagen-related peptide (5U/mL+5μg/mL). Graphs A-D show the percentage positive platelets (left y-axis) and median fluorescence intensity (MFI; right y-axis) for (A) Annexin V, indicating phosphatidylserine (PS) exposure, (B) PAC-1, indicating active conformation of $\alpha_{IIb}\beta_3$, (C) P-selectin, indicating α-granule exocytosis, and (D) LAMP-1, indicating lysosomal exocytosis. In graph E, the left axis shows median forward scatter (FSC) and the right axis shows the MFI of the platelet identification marker CD41. Data for all normal-sized platelets and all smaller platelets are shown for comparison. The bars indicate mean value, and error bars indicate the standard error of the mean (SEM), n=8. Repeated measures ANOVA followed by Bonferroni's post-hoc test was used for statistical comparison. */#/¤ = p<0.05, **/##/¤¤ = p<0.01 as compared to All normal-sized platelets/ DiIC$_1$(5)-positive normal-sized platelets/ DiIC$_1$(5)-negative normal-sized platelets.

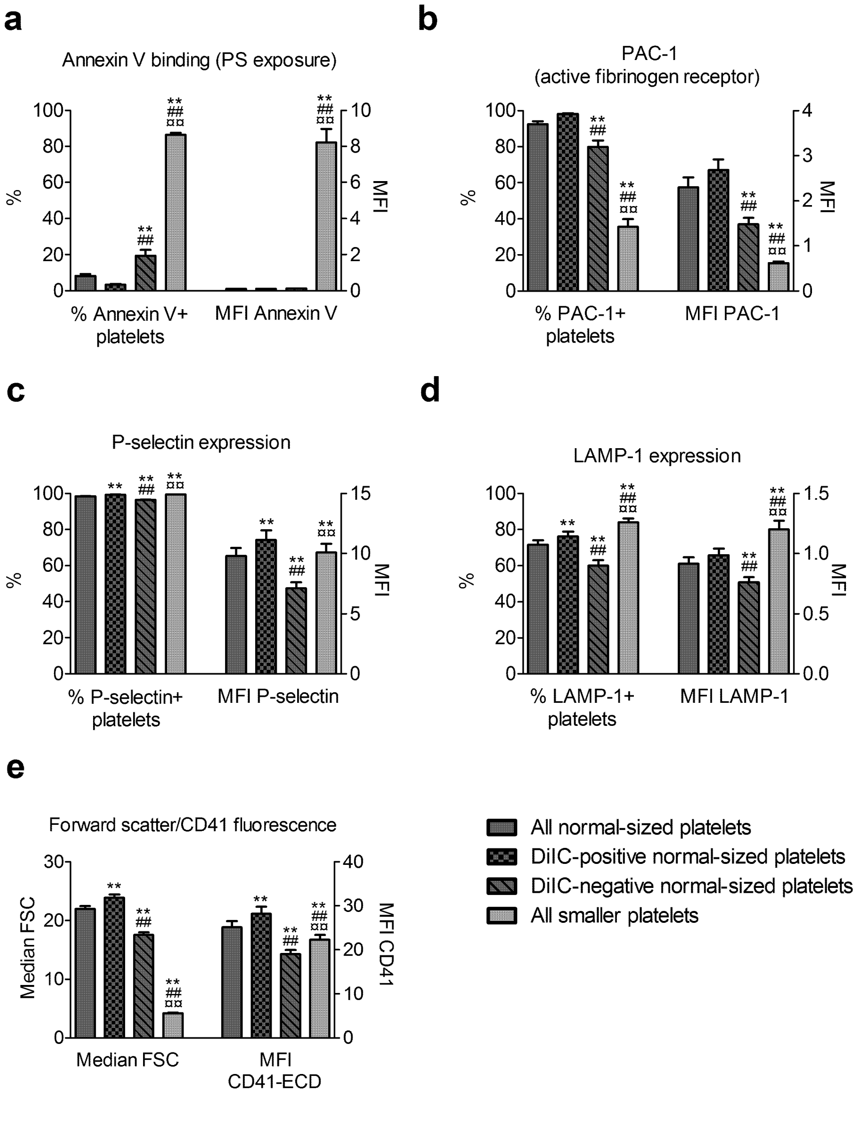

Supplemental Figure S7: Expression of platelet activation markers on normal-sized platelets level out at higher concentrations of platelet agonists.

Supplemental Figure S7A: Percent normal-sized platelets positive for the different platelet activation markers in response to increasing doses of thrombin or cross-linked collagen-related peptide (CRP-XL). The bars within the marker "NS" show no significant difference from the highest concentration investigated. Bars indicate mean value and standard error of the mean (SEM), n=5.

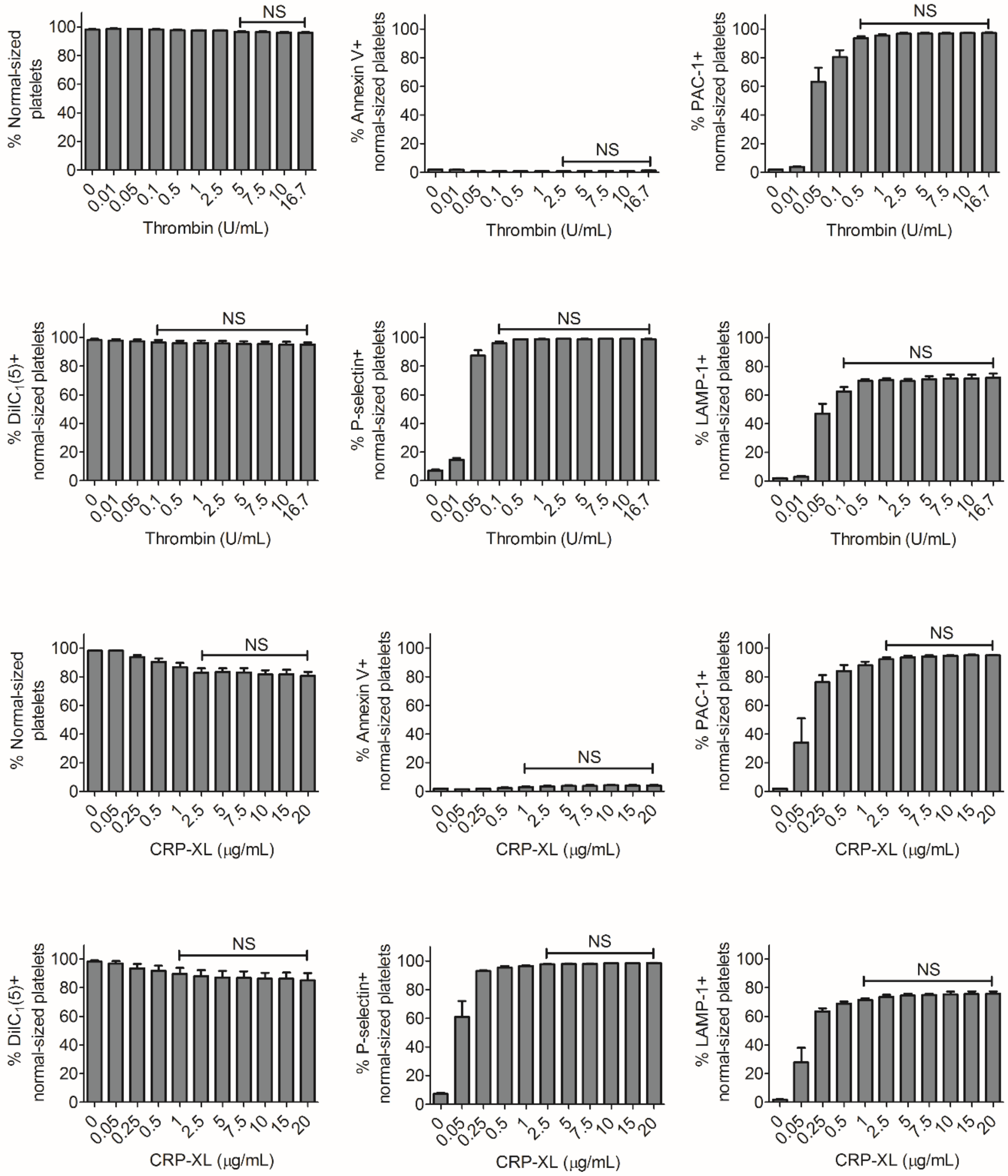

Supplementary information

Södergren AL and Ramström S: *"Platelet subpopulations remain despite strong dual agonist stimulation and can be characterised using a novel six-colour flow cytometry protocol"*

Supplemental Figure S7B: Median fluorescence intensity (MFI) of normal-sized platelets for the different platelet activation markers in response to increasing doses of thrombin or cross-linked collagen-related peptide (CRP-XL). The bars within the marker "NS" show no significant difference from the highest concentration investigated. Bars indicate mean value and standard error of the mean (SEM), n=5.

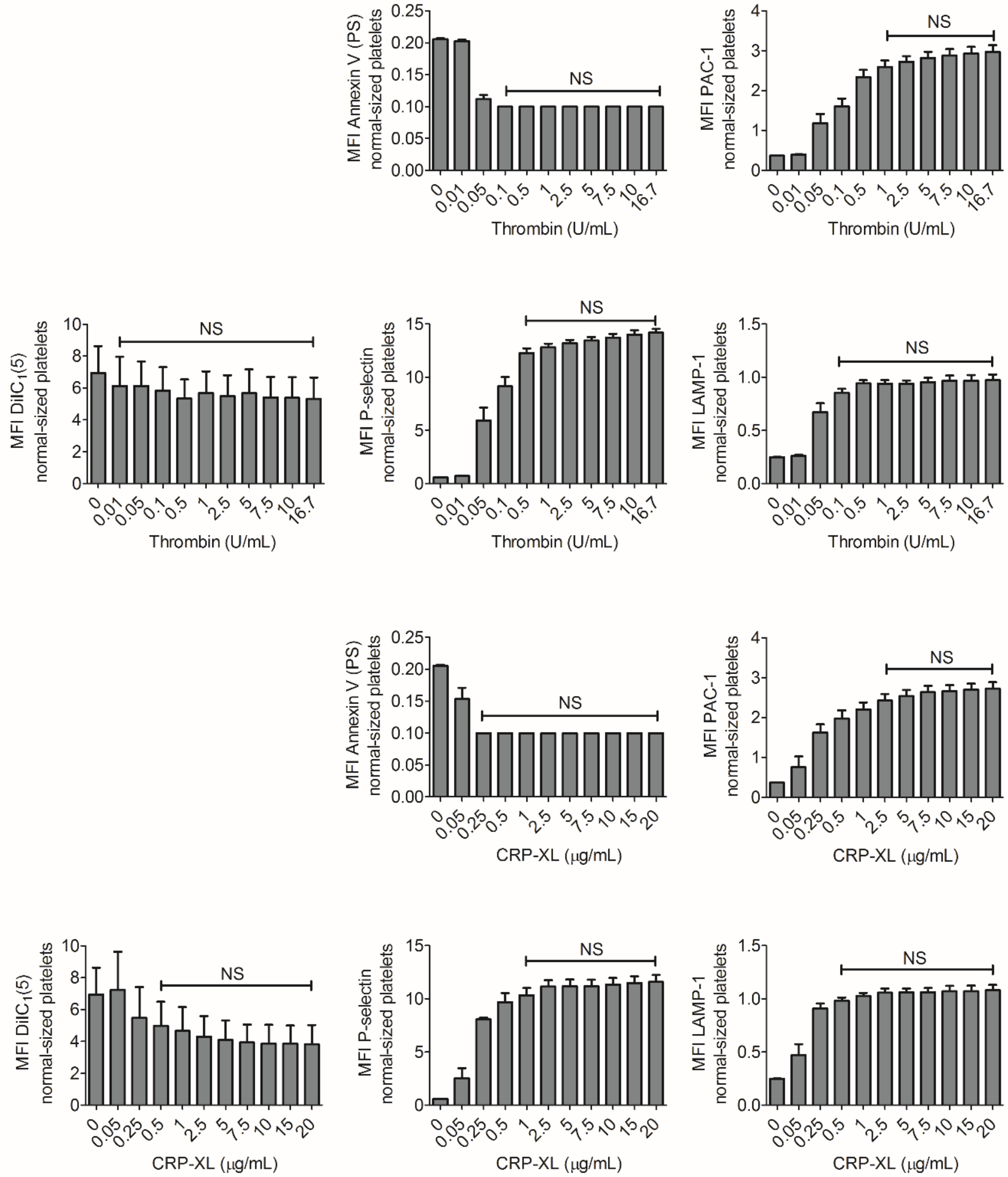

Supplemental Figure S7C: Percentage normal-sized platelets positive for the different platelet activation markers in response to 5U/mL thrombin or 5μg/mL cross-linked collagen-related peptide (CRP-XL) combined with increasing doses of CRP-XL or thrombin. The bars within the marker "NS" show no significant difference from the highest concentration investigated. Bars indicate mean value and standard error of the mean (SEM), n=5.

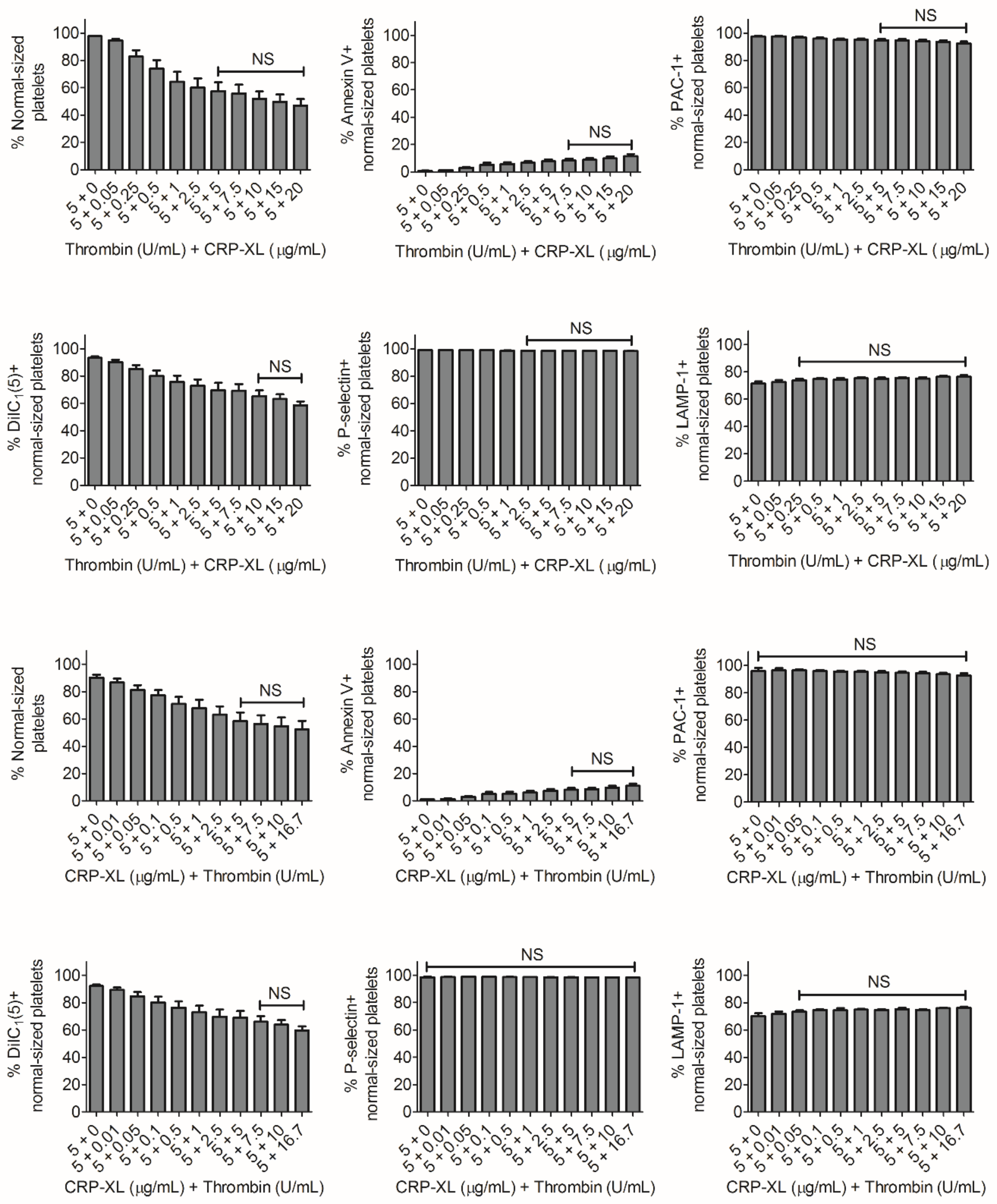

Supplemental Figure S7D: Median fluorescence intensity (MFI) for normal-sized platelets for the different platelet activation markers in response to 5U/mL thrombin or 5μg/mL cross-linked collagen-related peptide (CRP-XL) combined with increasing doses of CRP-XL or thrombin. The bars within the marker “NS” show no significant difference from the highest concentration investigated. Bars indicate mean value and standard error of the mean (SEM), n=5.

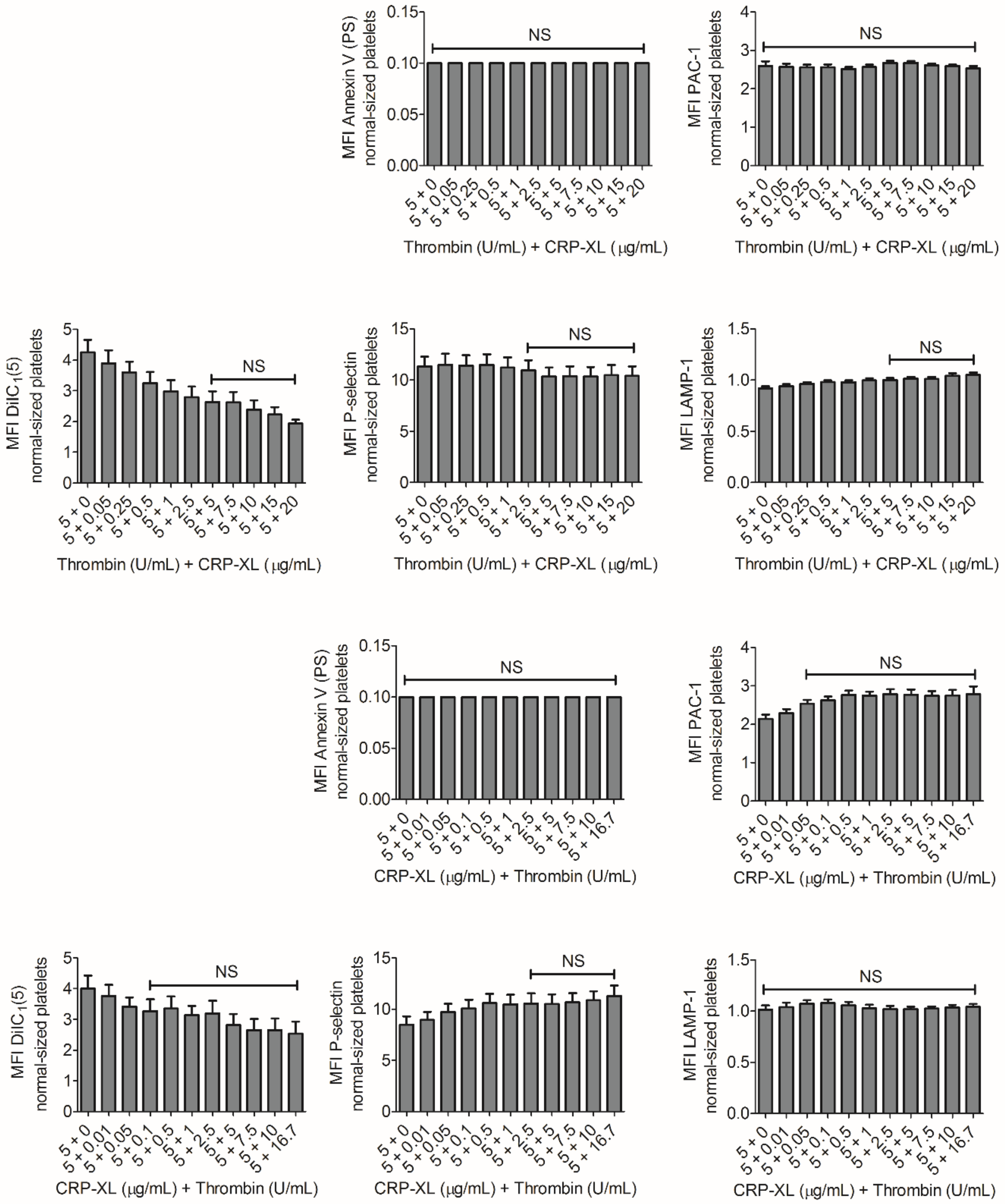

Supplemental Figure S8: Additional but slightly different platelet subpopulations form with prolonged activation times. Typical dot plots from samples activated with thrombin (5U/mL), cross-linked collagen-related peptide (CRP-XL; 5μg/mL) or thrombin+CRP-XL. Antibodies were added 10 minutes before the termination of activation.

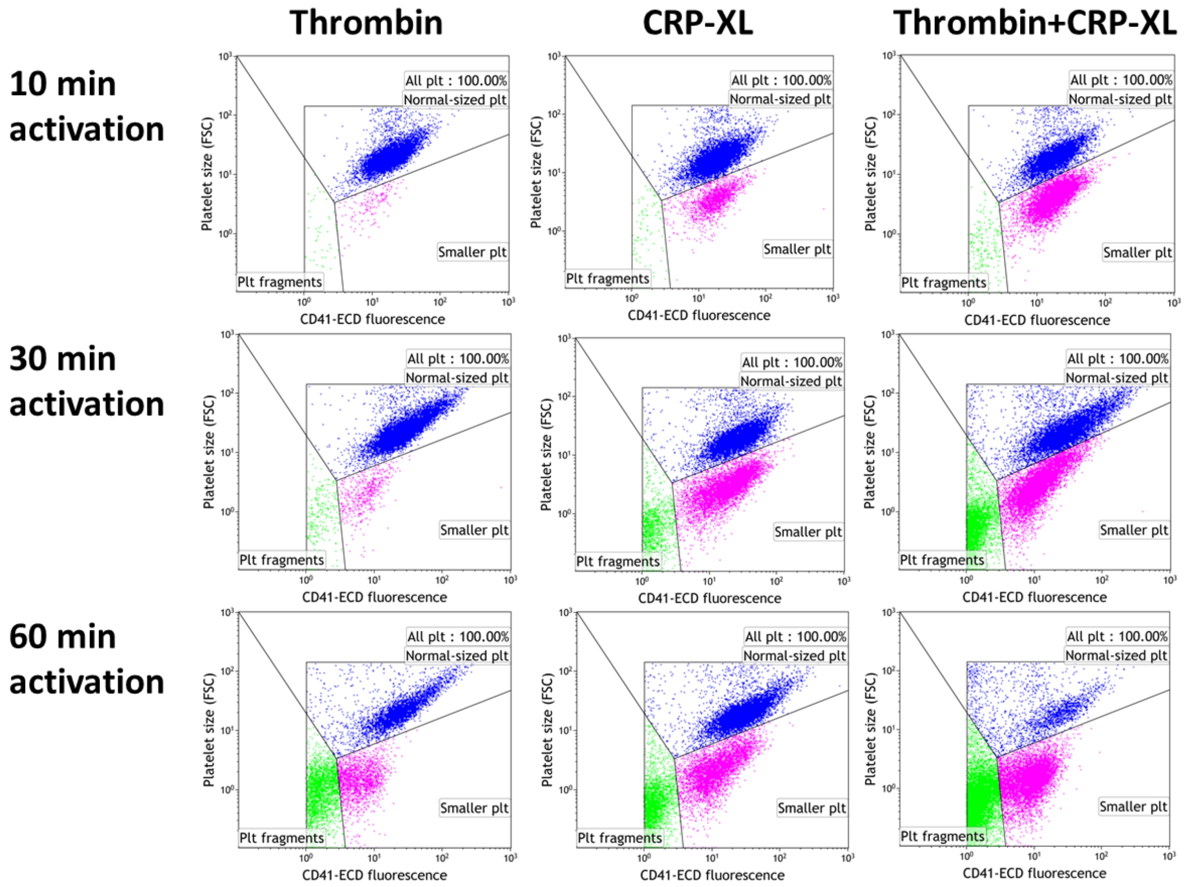

Supplemental Figure S9: Additional but slightly different platelet subpopulations form with prolonged activation times - median fluorescence intensity of platelet activation markers. Platelet samples were stimulated with buffer (Resting; black circles), thrombin (white squares; 5U/mL), collagen-related peptide (CRP-XL; 5μg/mL, black triangles, base down, broken line) or thrombin+CRP-XL (white triangles, base up). The graphs show exposure of platelet activation markers as median fluorescence intensity (MFI) for (A-C) Annexin V, indicating phosphatidylserine exposure, (D-F) PAC-1, indicating active conformation of $\alpha_{IIb}\beta_3$, and (G-I) DiIC$_1$(5), where fluorescence indicates retention of mitochondrial membrane potential. Graphs show mean value and standard error of the mean (SEM), n=5-13. Antibodies were always added 10 minutes before the termination of activation. * = p<0.05, ** = p<0.01 as compared to results after 10 minutes' activation. In resting samples, the number of smaller platelets and platelet fragments are low, hence these data should be interpreted with caution.

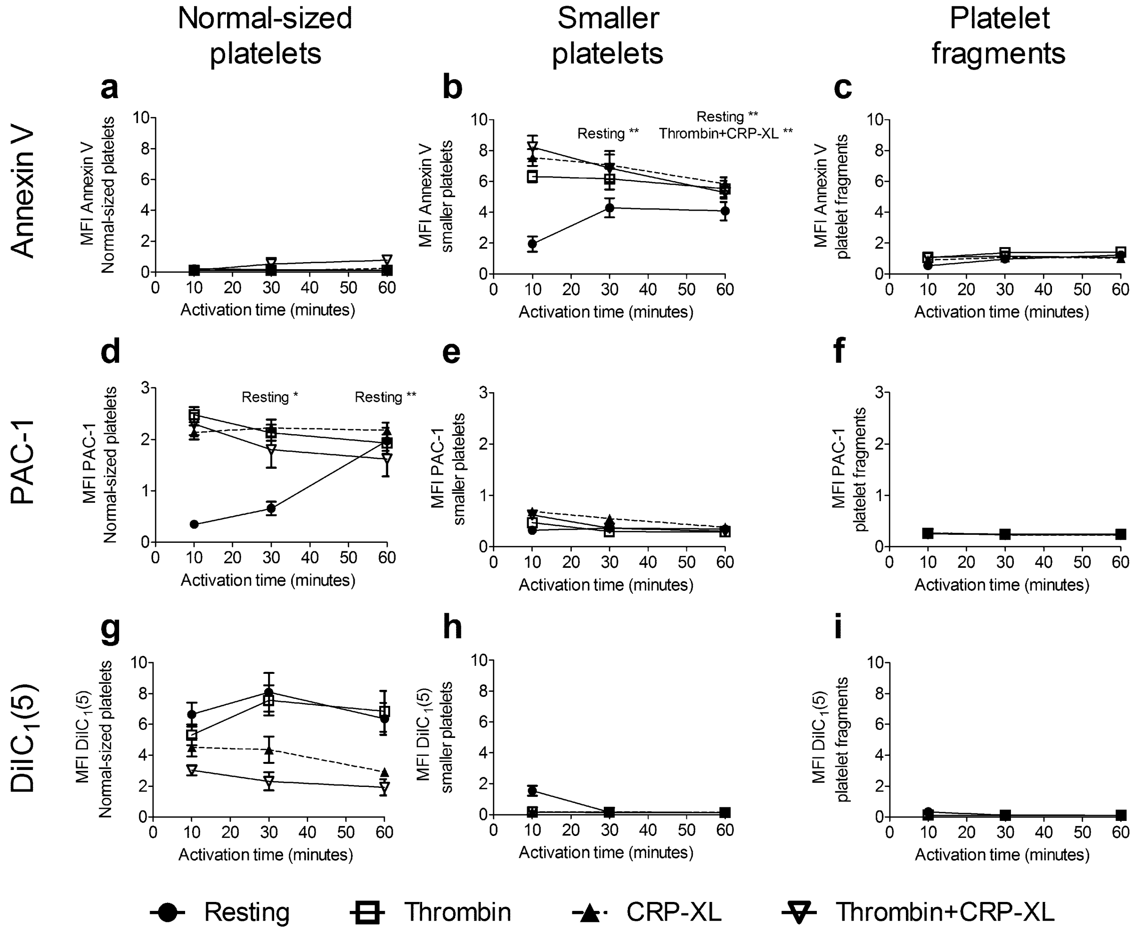

Supplement: Supplementary file 1 — Supplementary figures [file 41598_2017_19126_MOESM1_ESM.pdf]
